# A genetic algorithm optimization framework for the characterization of hyper-viscoelastic materials: application to human articular cartilage

**DOI:** 10.1098/rsos.240383

**Published:** 2024-06-26

**Authors:** Piers Allen, Sophie C. Cox, Simon Jones, Daniel M. Espino

**Affiliations:** ^1^ Physical Sciences for Health CDT, Department of Chemistry, University of Birmingham, Birmingham, UK; ^2^ School of Chemical Engineering, University of Birmingham, Birmingham, UK; ^3^ Institute of Inflammation and Ageing, University of Birmingham, Birmingham, UK; ^4^ Department of Mechanical Engineering, University of Birmingham, Birmingham, UK

**Keywords:** articular cartilage, dynamic mechanical analysis, finite element analysis, genetic algorithm, Prony series, viscoelastic

## Abstract

This study aims to develop an automated framework for the characterization of materials which are both hyper-elastic and viscoelastic. This has been evaluated using human articular cartilage (AC). AC (26 tissue samples from 5 femoral heads) underwent dynamic mechanical analysis with a frequency sweep from 1 to 90 Hz. The conversion from a frequency- to time-domain hyper-viscoelastic material model was approximated using a modular framework design where finite element analysis was automated, and a genetic algorithm and interior point technique were employed to solve and optimize the material approximations. Three orders of approximation for the Prony series were evaluated at *N* = 1, 3 and 5 for 20 and 50 iterations of a genetic cycle. This was repeated for 30 simulations of six combinations of the above all with randomly generated initialization points. There was a difference between *N* = 1 and *N* = 3/5 of approximately ~5% in terms of the error estimated. During unloading the opposite was seen with a 10% error difference between *N* = 5 and 1. A reduction of ~1% parameter error was found when the number of generations increased from 20 to 50. In conclusion, the framework has proved effective in characterizing human AC.

## 1. Introduction

Numerical methods have been used in various areas of study, for example, modelling the musculoskeletal system [1,2], fluid–structure interaction [3] and are ideally aimed at applications to clinical care [4]. This advance in utility has led to regulatory bodies such as the US Food and Drug Administration encouraging the inclusion of modelling data when submitting devices for approval [5,6]. Owing to the complexity of biological systems, many need to be represented at multiple scales, with some studies able to demonstrate the accuracy of the simulations [7–9]. These advanced models must not only represent the geometric structure but also its mechanical properties [10], leading to the inclusion of mathematical approximations. This requirement, however, leads to issues with the validation of the output from these models; they are used as surrogates for experimental work but their accuracy must be assured [11]. A reliable and effective model should include a strong link between simulations and experimental data [12].

When quantifying physical phenomena in numerical models, they are normally defined using partial differential equations and are often solved using finite element analysis (FEA) [13,14]. FEA has been used to facilitate the evaluation of experimental conditions, where analysis of the mechanics cannot be obtained either reliably or in a cost-effective manner. For example, toolboxes that generate models for FEA of the mitral heart valve [15] or the lumbar spine [16] enable the effect of geometric variables to be evaluated. While techniques to tackle anatomical variability and commercial software for structural optimization, e.g. ATOM by Abaqus [17], are available, there is scope to develop techniques for the optimization of material properties under representative physiological loading.

Sample variability, model robustness, validation bias and physiologically representative conditions are all factors that need to be accounted for in determining the accuracy of a material approximation, especially for biological tissues. There is a requirement for a large amount of experimental data in modelling systems [12] with some studies using optimization techniques to develop their material parameters [18]. This approach brings a need to separate training, testing and validation datasets to ensure robust validation of any optimization techniques used; techniques such as cross-validation [19] can be employed but this increases the data requirement to be effective.

Biological tissues exhibit material properties which can differ from standard engineering materials [20]. Articular cartilage (AC), for example, exhibits hyper-elastic [21] and viscoelastic [22] properties when dynamically loaded under conditions representative of physiological loading. AC has a reported range of material properties varying across 4–5 orders of magnitude depending on the method of testing used [20], induced stress [23] and frequency of loading [24]. Experimental techniques such as dynamic mechanical analysis (DMA) enable viscoelastic characterization of *ex vivo* biological samples under frequencies of loading relevant to their function within the body. However, this provides material properties within a frequency domain (such as storage and loss moduli). Applying such data to FEA simulations in a time domain, though challenging, is feasible via the characterization of a Prony series. For instance, a Prony series has been characterized by frequency-dependent storage and loss moduli for white and grey brain matter [25].

The aim of this study was to develop a framework for the automated characterization of time-domain hyper-viscoelastic properties from frequency-domain experimental measurements. The study is broken down into: (i) the gathering of experimental data through DMA; (ii) the automation of the modelling process including the creation of the FEA simulation, solving and evaluation; and (iii) the optimization of material property parameters. A key step is to allow the analysis of the models themselves to define how the parameters are altered on each sequential generation that is created. A case study of the application of the framework is presented for human AC, which is hyper-elastic and viscoelastic and as such a prime candidate with which to evaluate the framework. This study interfaces tissue biomechanics, machine learning and the automation necessary in numerical modelling to enable a genetic algorithm to be applied to the former for applications related to physiology and pathology conditions. This approach includes the distinction between training and validation datasets, as per practices in machine learning.

## 2. Methods

### 2.1. Experimental testing

**Figure 1.**
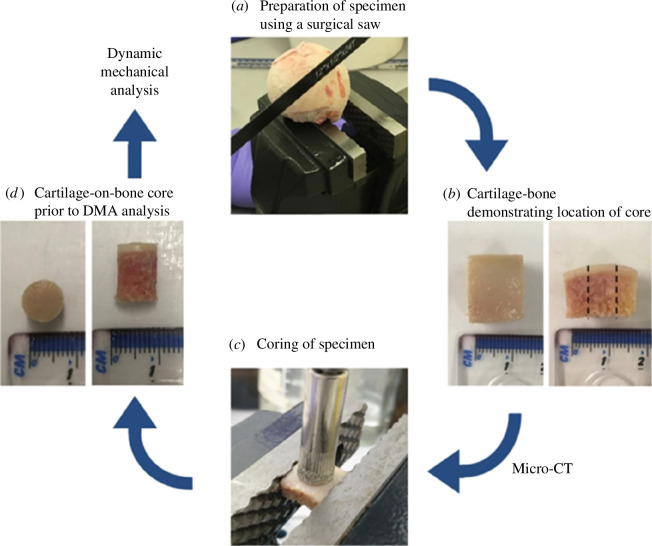
Flow diagram illustrating femoral head specimen preparation and coring: (*a*) preparation of specimen using a surgical saw, (*b*) example of cartilage–bone block prior to micro-CT analysis demonstrating where the core was taken, (*c*) coring of the specimen and (*d*) example of cartilage–bone core prior to DMA [22]. Reproduced from [22] under a CC BY4.0 licence.

The experimental dataset employed was that of Mountcastle *et al*. [22] where human AC specimens were tested. In total, five femoral heads were used with *n* = 16 unique test samples harvested (figure 1). Femoral heads were donated by patients who underwent surgery following a fracture of the femoral neck (ethical approval: East of Scotland Research Ethics Service, 11/ES/1044).

Femoral heads were stored at –80°C until 24 h before testing [22] and were defrosted [26] in Ringer’s solution. Each sample consisted of an 8 mm cartilage on bone core extracted using a diamond drill bit with the cartilage then being separated using a medical scalpel [23,27].

Experimental tests were performed using a Bose ElectroForce 3200 testing machine, controlled via WinTest 4.1 DMA software (Bose Corporation, Minnesota, USA; now, TA Instruments, New Castle, DE, USA). Two separate compressive loading sequences were performed, a quasi-static ramp and DMA. A preload of 0.02 N was followed by a quasi-static load at the rate of 3 N s^−1^, up to 61.6 N [21]. Then preconditioning loading cycles at 24 and 49 Hz were run for 1500 and 3000 cycles, respectively [28], consistent with AC requiring over 1200 [29] or 2000 [30] conditioning cycles for cyclic testing. Finally, a frequency sweep was completed between 1 and 88 Hz [28].

DMA was performed as described by Lawless *et al*. [31]. Briefly, the viscoelastic material was characterized by its complex stiffness $\left(k^{*}\right)$ using the magnitudes of the load and displacement dataset lengths, following a fast Fourier transform, for each frequency. A shape factor $\left(S_{F}\right)$ for the test specimen’s geometry, its complex modulus $\left(k^{*}\right)$ and the phase lag ( δ) between load and displacement waveforms are then used to determine the storage $\left(E^{\prime }\right)$ and loss $\left(E^{\prime \prime }\right)$ moduli (equations (2.1), (2.2)):


(2.1) $$E^{\prime }=\;\frac{k^{*}\cos\left(\delta \right)}{S_{F}},$$

(2.2) $$E^{\prime \prime }=\frac{k^{*}\sin\left(\delta \right)}{S_{F}}.$$

### 2.2. Automated modelling system structure

**Figure 2.**
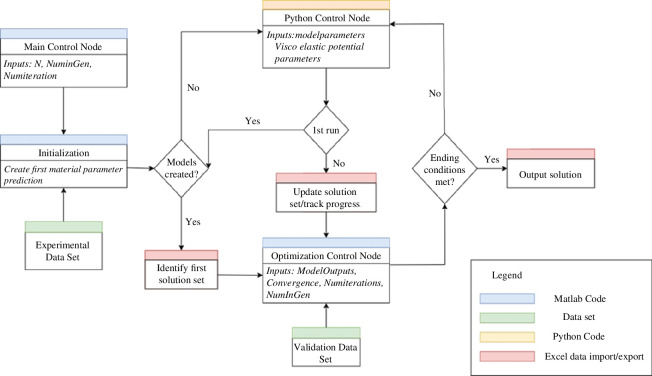
Flow chart representation of the control structure for the automation system, with boxes representing code/data and diamonds representing questions the system asks through its iterations.

Two cyclic control structures linked optimization with the modelling software and provided the primary automation. The modelling procedure is controlled through the Python node apart from the output analysis which is performed using Matlab (The MathWorks Inc., Massachusetts, USA). The optimization of the material parameters is completed using a separate Matlab script (figure 2). Before the cycle starts, an initial optimization takes place to create the first parameter set.

These two components work together in an overarching cyclic system that completes a loop upon every cycle of the machine learning algorithm. The Matlab code produces the parameters that comprise the current dataset which is then passed through to the Python node. This Python node then produces, applies loads to and solves models for each of the parameters and outputs a dataset of material properties. This is then passed through fitness evaluation in the Matlab scripts and then a new parameter set, of each child, is produced starting the cycle again until ending conditions are met. A visual representation of the overall structure of the system and how these two components work together is laid out in figure 2. As part of the optimization procedure, the Python control node is run on every generational cycle of the algorithm to create and solve the models. It provides the simulation data of the material properties used as part of the ‘fitness’ evaluation of each child of that generation’s data inputs.

The total dataset used has 168 unique data tuples which include frequency (Hz), storage modulus (MPa) and loss modulus (MPa). The dataset is then divided into 128 datasets for training and 40 data points for validation, which are split at random on each run. This first optimization is performed using an interior point technique on the training dataset. The validation dataset is used by the Matlab script to evaluate the efficacy of the produced models. The Matlab script requires three user inputs (excluding predefined variables defined below): ‘*N*’, the degree of the approximation; ‘NumIteration’, the maximum number of generations the optimization will complete; and ‘NumInGen’, the number of models generated per generation (minimum limit of 100, as the system requires several models to compare for each generation). A limiting factor of this system is the time complexity, which is controlled by the three variables above.

The Python control node is self-contained and modular; thus, its code does not change per iteration, and only the required inputs are altered. Two-dimensional AC models (electronic supplementary material, figure S1) are created which, briefly, consist of the shape, conditions of loading, physical properties of the material, and set-up for FEA; with further details on meshing and modelling provided in §2.3. They are generated from a defined set of parameters (provided by the optimization control node) and then sequentially solved for each combination, providing a range of models to be analysed for efficacy. The lack of required deviations in the code allows for seamless running without the need for user input at this stage. Displacement values are extracted from the solved jobs and passed through to a Matlab analyser script that compares them to the experimental validation set, allowing the models to control the progression of the material parameter development. After every generation of models, parameters are stored in the backup to track property progression.

In the optimization node, initially, the model outputs are sorted and compared with an evaluation dataset that is composed of three separate displacement profiles representing the ramp test, and then the load and unloading during the sinusoidal loading sequence. It is then checked to establish whether the ending conditions have been satisfied which could include a number of factors such as maximum cycles, minimal error and no error change. If not, the optimal solutions are then taken and passed into the optimization module.

A genetic algorithm is used to progress the development of the parameters during optimization. The mutator uses crossover, Gaussian distribution alteration and random mutation to create many possible parameter compositions. These are evaluated against a viability check, and then the defined number of models (NumInGen) is passed back to the Python control sequence. The next generation of models created is a combination of the top results from the previous generation, outputs from the mutator and random possibilities. All code is stored in GitHub and archived within the Zenodo repository [32].

### 2.3. Finite element analysis

FEA has been performed using ABAQUS 6.14 (Dassault Systèmes Simulia Corp., Providence, RI, USA), controlled using the inbuilt functionality of Python script macros. AC was modelled as a cross-sectional segment of the cylindrical test samples with dimensions matching the sample cores that were tested experimentally. The geometry is set to 8 mm in length (mean), and 1.5 mm in height taken from sample thickness data; as per [21]. Three options of mesh were defined at 100, 150 and 200 μm, providing mesh sizes between 200 and 800 elements to test variation in error among mesh conditions. To mimic physical testing two boundary conditions are applied to the model. The base was completely restricted in displacement and rotation, and the top was restricted to only move along the *y* axis, which has been defined as being perpendicular to the surface of AC test samples and aligned with the direction of load (*–y* axis; electronic supplementary material, figure S1). These models are used as input files for the purpose of the automation. For every possible combination of material properties proposed, an individual model was created and solved with the above definitions. For each iteration during the optimization, 1000 viscoelastic potential parameter solutions were created alongside a fixed Ogden hyper-elastic definition.

For each simulation, a uniform load was applied across the top surface of the geometry of 0–1.7 MPa to mimic the experimental testing performed. This was applied in two sequential stages lasting for 1 s. The first section performed a simulated ramp compression step loading from 0 to 1.225 MPa. The second portion of the sequence performed a sinusoidal loading step calculated via a time-dependent multiplication factor (*a*), frequency of loading ( ω ), initial step time (*t*_0_) and current step time (*t*) (equation (2.3)):


(2.3) $$a=1.225+0.493\cdot \cos\left(2\cdot \pi \cdot \omega \cdot \left(t-t_{0}\right)\right).$$

This amplitude produced a representative 1 Hz sinusoidal stress of 0.7 to 1.7 MPa which is representative of values observed physiologically [33] and in DMA tests. Loading was applied uniaxially to the top face of the model with the displacement measured.

### 2.4. Material characterization

To simulate AC, a hyper-viscoelastic approximation was used in Abaqus which included: a Poisson’s ratio of 0.45 [34]; a Prony series (§2.4.1); and an Ogden model [35] for the hyper-elastic approximation. The hyper-elastic, strain-energy potential function is simplified to equation (2.4) and includes *N* in this case the order of the model, *µ*_*i*_ is a shear term, *α*_*i*_ is a dimensionless material constant and *λ*_*i*_ are principal stretch values (evaluated automatically by Abaqus):


(2.4) $$U=\sum_{i=1}^{N}\frac{2\cdot \mu_{i}}{\alpha_{i}^{2}}\left(\lambda_{1}^{\alpha_{i}}+\lambda_{2}^{\alpha_{i}}+\lambda_{3}^{\alpha_{i}}-3\right).$$

The values used for the Ogden approximation were taken from [21]; other hyper-elastic models can be modelled through minor alterations to the Python data import file.

#### 2.4.1. Viscoelastic model

The generalized Maxwell model, used here, combines a singular main elastic branch with *N* spring–dashpot branches. This was implemented as a Prony series to represent the time-domain viscoelastic response for a material modelled:


(2.5) $$\mu \left(t\right)=G_{\infty }+\sum_{i=1}^{N}g_{i}\exp\left(-\frac{t}{t_{i}^{\prime }}\right),$$

where $\mu \left(t\right)$ is the time-dependent relaxation modulus, $G_{\infty }$ is the equilibrium modulus and $g_{i}$ and $t_{i}^{\prime }$ are the spring relaxation modulus and relaxation time of the Prony series for *N* spring–dashpot pairs or frequency delays. The relaxation modulus $\mu \left(t\right)$ can be expressed as a discrete set of exponential decays [25] and the complex modulus $u^{*}$ is defined as


(2.6) $$u^{*}\left(j\omega \right)=G_{\infty }+\sum_{i=1}^{N}g_{i}\frac{t_{i}^{\prime }j\omega }{1+\;t_{i}^{\prime }j\omega },$$

where ω is the angular frequency and $j=\sqrt{-1}$ . This expression is derived as the Laplace form (equation (2.5)). Thus, the Prony series representations of storage (equation(2.7)) and loss (equation (2.8)) modulus in terms of frequency can be defined:


(2.7) $$u^{\prime }\left(\omega \right)=G_{\infty }+\sum_{k=1}^{N}g_{k}\cdot \frac{{\left(\omega \tau_{k}\right)}^{2}}{1+\;{\left(\omega \tau_{k}\right)}^{2}},$$

(2.8) $$u^{\prime \prime }\left(\omega \right)=\sum_{k=1}^{N}g_{k}\cdot \frac{\left(\omega \tau_{k}\right)}{1+\;{\left(\omega \tau_{k}\right)}^{2}}.$$

The dynamic moduli, $u_{i}^{\prime },\;u_{i}^{\prime \prime }$ , at a given frequency, $\omega_{i}$ , with $u^{\prime }\left(\omega_{i}\right)\;\text{and}\;u^{\prime \prime }\left(\omega_{i}\right)$ being the respective predicted values are put through a minimization equation using the original generalized Maxwell model (equation (2.9)):


(2.9) $$\min_{g,t\in R^{N}}F\left(g,\;\tau \right)=\sum_{i=1}^{N}\left({\left(\frac{u^{\prime }\left(\omega_{i}\right)}{u_{i}^{\prime }}-1\right)}^{2}+{\left(\frac{G^{\prime \prime }\left(\omega_{i}\right)}{G_{i}^{\prime \prime }}-1\right)}^{2}\right).$$

The optimization problem solves for so-called Prony parameters ( g , $G_{\infty }$ , *N*, $\tau_{k}$ ) so that they simultaneously satisfy experimentally generated storage and loss modulus calculation from DMA tests, where ***g =*** ( $g_{1},\ldots ,g_{N})$ ) and τ = ( $\tau_{1},\ldots ,\tau_{N})$ ) for the defined series length, *N*.

The parameters were determined using a two-stage optimization process within the overall system. They enforce that sequential time values $\tau_{i}$ are always increasing in value and that the summation of all the equilibrium shear modulus values summate to less than 1 (equations (2.10)–(2.12)):


(2.10) $$\tau_{i}\;\leq \;\tau_{i+1}\;\;\forall \;i,$$

(2.11) $$\;\;\forall \;k\sum_{k=1}^{N}g_{k}\leq \;1,$$

(2.12) $$\;0<\;g_{k}<1.$$

### 2.5. Python control structure

As thousands of FEA models were solved during a single system run, the computational load of the design and solving procedure was minimalized. For each iteration of the automation, one FEA model was solved, which sequentially created and then solved all defined models. Inputs into the control sequence were defined through two Excel documents controlling the model parameters that are common to all models and then a secondary document that contains the material parameter definition. The first spreadsheet (sample values in table 1) controls the model construction and is unique to the individual overall model being simulated. The file also includes the hyper-elastic material coefficients (table 2). In this example, only one selection of coefficients is supplied; however, akin to loads/meshes, multiple sets of coefficients may be provided, and all combinations of simulations will be produced; the caveat being that all approximations must have the same number of material parameter values for each coefficient set, in this case six points, as a third-order model is used with $\alpha_{i}$ and $\mu_{i}$ (equation (2.4)).

**Table 1.** Example of the required used defined parameters that are global variables to all the models produced. The actual format required is available via the code provided [32]. The dimension values all determine the geometric structure of the object undergoing compression.

dimension values							
GridSpaceX1	GridSpaceX2	XOrigin	maxWidth	GridSpaceY1	GridSpaceY2	YOrigin	maxHeight
0	0.008	0.004	80	0	1e-03	5e-04	80

loading/material/meshing values							
elastic modulus		Poisson ratio		load magnitude (MPa)		mesh density	
1e+09		0.45		1 255 000		1.5e-04	

**Table 2.** Ogden material approximation parameter set used.

$\mu_{1}$ (Pa)	$\alpha_{1}$	$\mu_{2}$ (Pa)	$\alpha_{2}$	$\mu_{3}$ (Pa)	$\alpha_{3}$
26 133 000	2.7190	12 922 000	3.9960	13 227 000	1.504

**Figure 3.**
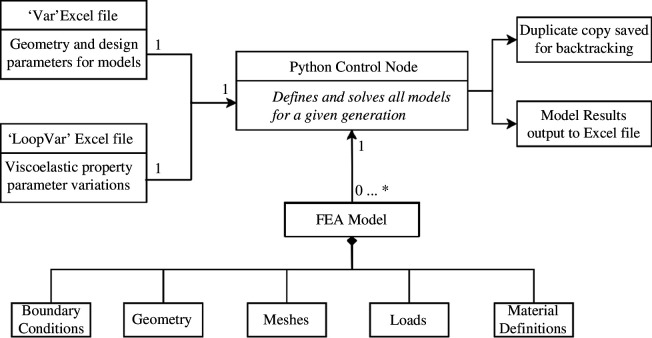
Python control node class diagram. The bottom set of boxes represents the different portions of the model creation system that are currently implemented. This can be increased with any other requirements.

A secondary file stores the current iteration of the Prony coefficients tested, updated on each iteration and which can contain anywhere from 10 to 500 possible combinations depending on the limitations placed by the user on the algorithm. Each variation is backed up so that backtracking and analysis of a previous solution is possible; i.e. how variables change across generations can be tracked (figure 3).

The spreadsheets are converted to Python variables, upon every iteration of new values, and passed into the main class of the Python script. The Excel spreadsheets are converted using a Matlab functionality called ‘fprintf’ which allows direct text to be written in a notepad format. Once converted to notepad, the file can be read-in by the Python script as a variable file if formatted correctly. The process for the evaluation and creation of models is defined in table 3 alongside a class diagram depicting its relational schema (figure 3).

**Table 3.** Model creation and retrieval of results as performed in Python’s interaction with Abaqus.

creation of the generation’s models
1. reading in of required variables (2 Excel spreadsheets)
2. set limitations on the number of models to be created based on the number of variations in the variables
3. create a new blank model and job for each combination of variables
4. loop through combinations defining model values (loads/material coefficients/meshes etc.) based on variations

solving and results collating
1. model database saved for tracking progress
2. individual jobs looped through and solved
a. nodal results for displacement and load magnitude saved
b. results collated into correct folders alongside other tracking information: model database for the current generation, variables file and all combinations of variables
3. Abaqus module closed as analysis occurs within Matlab and to reduce computational usage

### 2.6. Matlab optimization control sequence

The main cycle components for control (figure 2) are model evaluation and optimal solution identification, dataset permutation, genetic mutation, evaluations for model creation, send to modelling node and restart. The process uses an interior point optimization [36] technique to generate an initial starting point for the genetic algorithm, optimizing the problem via solving sequential, approximate minimization problems. Mathematically, if the original minimization problem is defined in equation (2.13), the approximate solutions are defined as equation (2.14) which has the introduction of as many slack variables $s_{i}$ as there are inequality constraints $g\left(x\right)$ . The aim is that as *µ* trends to 0 so does the result of *f*(*x*).


(2.13) $$\min_{x}f(x),\;st:\;h(x)=0\;and\;g(x)\leq 0,$$

(2.14) $$\forall \;\mu >0,\;\min_{x,s}f_{\mu }\left(x,s\right)=\min_{x,s}f\left(x\right)-\mu \sum_{i}\ln\left(s_{i}\right),\;st:\;s\geq 0,\;h\left(x\right)=0\;and\;g\left(x\right)+s=0.$$

The limits, *h* and *g*, were set to the Prony limits (equations (2.11) and (2.12)). A random seed, defined by the computer time, is used to define the initialization point of the algorithm with 100 iterations performed to get a range of solutions from unique starting points. The solution set from this first step is what provides the automatic loop with its initial parameter starting point.

#### 2.6.1. Main optimization sequence overview: genetic algorithm

**Figure 4.**
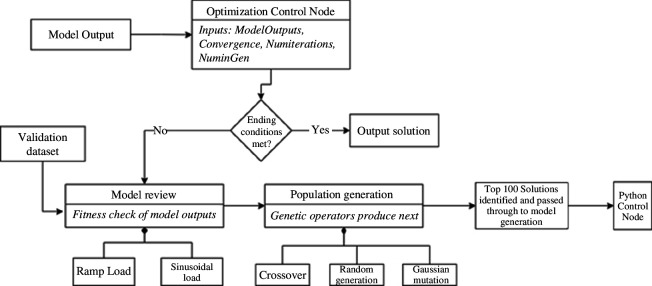
Optimization sequence control structure flow diagram representation.

Genetic algorithms mimic natural selection. The main components of any genetic algorithm are the genetic operators and the fitness check. There are two fitness checks that occur to develop the parameter solution. One is based on calculating the error produced by the Prony series equation and the other uses the model output and compares that directly to the experimental dataset. This allows a larger population to be created without hindering the overall efficiency of the system The cycle of an individual iteration is seen in figure 4.

#### 2.6.2. Genetic operators

Upon each generation, the new population is created using several genetic operators which are crossover, Gaussian mutation and random generation, with each of them producing a third of the new generation. The population amount is defined in the code as ‘numinpopulation’ but is not controllable by the user-defined variables. Owing to the limits required by the solution (equations (2.11) and (2.12)) all children created are checked for validity; this ensures the time values are all sequential and the *g* values all sum to 1.

Both single and *K*-point crossovers are performed with a 50:50 split of population production. Single point crossover is performed by producing a uniformly distributed random number between one and the length of the solution (2 × *N*) + 1 where *N* is the number of series expansions. The parent solutions are split at this point and then recombined to form two new unique solutions. *K*-point crossover has the same theory, but instead of generating one crossover point, a user-defined number of crossovers take place.

#### 2.6.3. Equation-based fitness check

The evaluation based on the equation is performed as soon as the dataset for any given generation is created, ranging from 1000 to 100 000 permutations of the parameter set. A dataset of 128 unique experimental data points was used to compare against the parameter possibilities. The validation dataset of 40 points is stored as storage and loss moduli (equation (2.9)) and the parameter values for $g_{i}\;\text{and}\;\tau_{i}$ , these data points can be substituted in and thus a difference computed. This is then averaged across all the data points and an ‘error’ value is produced for each possible solution. Owing to the volume of checks that are being computed growing exponentially and being defined by *N* (degree of approximation) multiplied by dataSize (number of comparison points) and GeneticMutations (number of permutations in each generation), ensuring this calculation was computationally efficient was necessary. The fitness check reduced the number of solutions already computed and hence the number of models generated to the value of ‘NumInGen’.

#### 2.6.4. Model-based fitness check

The values that are used to evaluate which model outputs to continue with are load, time and displacement, split into three portions: ramp test and upper/lower loading profile of sinusoidal load. The latter required time/displacement data to be converted into a hysteresis loop, representing the entire time period. The hysteresis for every simulation is then split into its upper and lower curve structure. This allows the parameter solutions to be evaluated for how they represent the different loading and unloading material reactions seen in the experimental data. The error values reported are averaged percentage differences across every displacement/force data point produced by the model.

**Figure 5.**
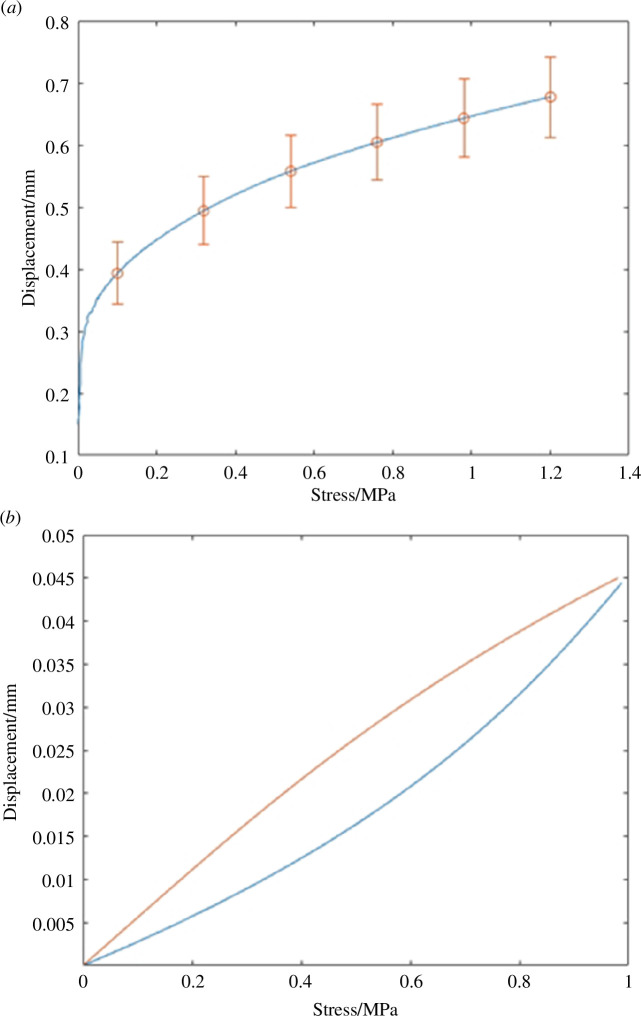
Experimentally obtained data. (*a*) Mean ramp test data from six experiments (red circles, with standard deviation shown as error bars) curve-fitted to a logarithmic function (blue line) to be used as part of the validation of the models produced in each generation. (*b*) Example hysteresis dataset produced from the validation portion of the experimental data and fitted to a logarithmic and exponential function for the upper (red line; loading stage of the cycle) and lower (blue line; unloading stage of the cycle) lines, respectively. These curves are produced on the initialization of the system and are then used throughout remaining unaltered for consistent fitness evaluation. Note that both graphs plot displacement (mm) against stress (MPa); where the latter was led experimentally via load control.

The dataset used for validation of the ramp tests was gathered by performing six separate ramp loading sequences without the additional DMA. The six tests were then averaged to provide an overall approximation of the experimental ramp data. To ensure both datasets (experimental and model) are comparable the validation data are approximated by a logarithmic representation (figure 5).

The evaluation of the sinusoidal load starts with the model output. this must first be normalized to the lowest displacement value of 0 and then split into its upper and lower loop loading profiles. The experimental data were taken from the validation set of DMA experiments referenced earlier.


(2.15) $$y=a_{1}\cdot \log\left(b_{1}\cdot x\right),$$

(2.16) $$y=a_{2}\cdot e^{\left(b_{2}\cdot x\right)}.$$

The upper half was approximated using a function of the form displayed in equation (2.15) and the lower half with equation (2.16), with *a*_1_, *b*_1_ and *a*_2_, *b*_2_ being the optimized variables per equation set, respectively. An example hysteresis validation set is shown in figure 5*b*.

## 3. Results

The results presented include 30 unique simulations completed via the automation system. They are composed of three *N* (order of approximation) values of 1, 3 and 5, for the Prony series, and two iteration lengths of 50 and 20 split into the *N* values at six 50 runs and four 20 runs (table 4). All initial solutions were derived using the native Java rand function using the current (at the time of simulation initialization) computer clock as the seed to ensure no initial bias gave one simulation benefit over the others.

**Table 4.** Final mean error values of each simulation averaged across the 30 repeats with s.d. included. The error values correspond to the difference between the resultant approximations and the experimental dataset averaged across multiple simulations. Done for (A) 50 iterations and (B) 20 iterations.

	ramp		lower (unloading)		upper (loading)	
*N*	mean	s.d.	mean	s.d.	mean	s.d.
(A)	50 iterations of the genetic algorithm (% error)					
1	16.95	0.68	24.6	0.28	20.98	0.81
3	15.79	0.02	19.89	1.80	21.69	1.51
5	15.79	0.02	15.74	0.75	30.69	0.03
(B)	20 iterations of the genetic algorithm (% error)					
1	17.75	1.94	24.59	0.19	21.62	1.30
3	15.79	0.03	19.82	1.95	24.02	4.14
5	15.84	0.04	16.09	0.60	30.73	0.09

From each simulation error values for each ramp, upper hysteresis and lower hysteresis load values were taken on every iteration using the optimal parameter set at that point in progression. The final output error across the entire loading curve (i.e. the ‘mean error’) was calculated and can be observed in figure 5. The final mean error values were in the range of 15–30% regardless of whether 20 or 50 repeats were solved (table 4).

**Figure 6.**
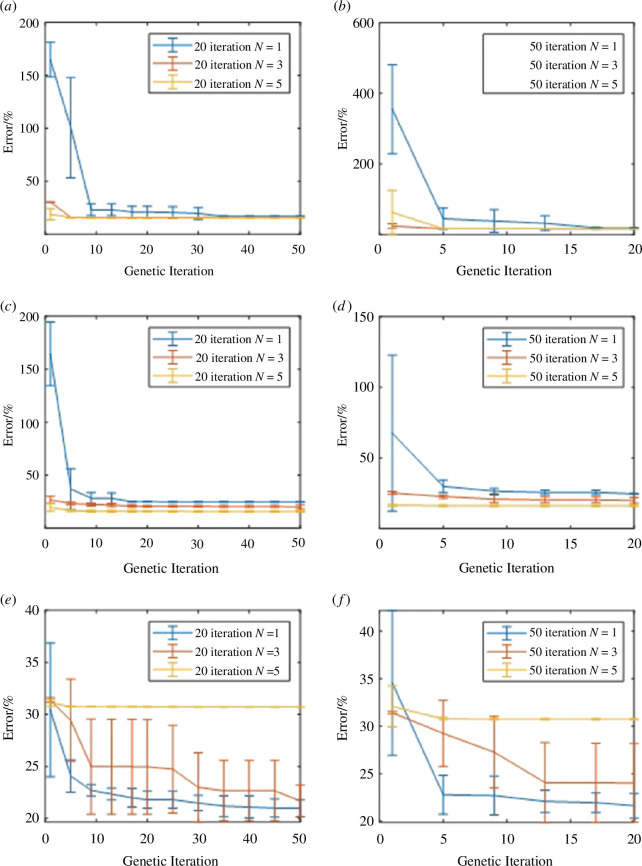
Error analysis for all 30 simulations as per the algorithm progression. (*a*) 50 iteration ramp error output, (*b*) 20 iteration ramp error output, (*c*) 50 iteration lower hysteresis error, (*d*) 20 iteration lower hysteresis error, (*e*) 50 iteration upper hysteresis error and (*f*) 20 iteration upper hysteresis error.

The ramp load error (figure 6) calculation. For *n* = 1 the final parameters produced on average more error after both 20 and 50 iterations compared with *n* = 3 and 5. In this result set, the produced error when *N* was defined at 3 and 5 was 2% smaller at *n* = 1 after 20 iterations and still 1% smaller after 50 iterations. An additional feature of both *n* = 3 and 5 was that the deviation among the repeats was negligible in both tests with a much larger deviation seen in *n* = 1 results. There is also a negligible difference between the datasets produced for *n* = 3 and 5.

Figure 6*c,d* presents the lower or unloading portion of the hysteresis loop at 50 and 20 iterations, respectively. The simulations for *n* = 1 perform consistently worse across both sets of simulations with it resulting in around 5% more error than *n* = 3 and 10% more than *n* = 5. Comparing *n* = 3 and 5, both versions plateau after around 20 iterations with *n* = 5 performing around 5% better. The value *n* = 3 does continue to reduce the error until 50 iterations but at a much more gradual pace demonstrated by the final error value having a large standard deviation.

Figure 6*e,f* displays the loading portion of the hysteresis loop at 50 and 20 iterations, respectively. When *n* = 5, the error was unable to be improved from the initial iteration in both the 20 and 50 iteration runs and produced an overall error value ~10% larger than *N* values of 1 and 3. Given that the standard deviation was very small in both 20 and 50 iterations it shows that this occurred for every *n* = 5 simulation. It is seen that the error continued to be reduced up until the point of 50 iterations for both *n* = 1 and *n* = 3 giving an improvement of 1% for *n* = 1 and 3% for *n* = 3. The convergence across the simulation repetitions also improved as the iteration progressed as shown by the deviation bar values reducing as the later iterations are reached.

For completeness, the Prony series characterized for femoral head human AC, for *n* = 1, 3 and 5 (20 and 50 iterations) are provided in the electronic supplementary material (tables S1–S3). Results from six simulation sets are presented.

## 4. Discussion

The term ‘optimization’ is often misrepresented, being used when parameters are purely varied without the evaluation of a true optimal solution [37,38]. For example, varying Young’s modulus over 10 values to find a ‘best’ value is not an optimized solution, but the best of 10 proposed solutions: a common misconception. Further, where a variable range is constrained by a user in this manner, solutions can be stationed within a local minimum rather than the global minima. A related pitfall is overfitting [39], where the solution chosen is specific to a particular dataset and thus might be unsuitable when used to predict future observations.

The framework developed in this paper has used an automated, optimization approach to characterize the hyper-viscoelastic properties of human AC from the femoral head. Using the model outputs as the driving force for each generation’s progression allows not only the material approximation to be a factor for the parameters but also the direct comparison of an experimental result to a model’s response. In total, 30 sets of simulations of the automation system with varying initialization parameters were run, to test the effectiveness of this technique with the aim of showing consistency and effectiveness of said framework. Furthermore, a methodology has been made available which enables data obtained under a frequency domain to be available for time-domain analysis; optimizing material characterization [32].

The use of numerical methods and modelling has seen an uptake in biomedical research over the last 20 years; however, development is still required as demonstrated by Bhattacharya *et al*. [40]. Within this field, an important component is the validation of said numerical methods [41]. Cyclic optimization of material parameters has been used elsewhere [42]; in our study, we present a technique which focuses on validation intrinsic to the characterization of the material properties, and the process is automated. This unique approach allows the simulation of actual experimental test results to be a part of the fitness evaluation. There could be scope, in future, to replace input data tuples from experimental testing with histomorphological properties for subchondral bone, as these can be correlated to the storage and loss moduli for AC [43]; as per figure 1, it is noted that micro-CT was performed on all samples used in this study [22], though these data are not currently incorporated within the code. The application of genetic algorithms to optimization has been demonstrated [44,45] but could clearly have greater use within the biomedical field.

The framework has been evaluated for human AC and has enabled time-domain parameters to be obtained for a hyper-viscoelastic model, from frequency-domain experimental testing. It was seen in all situations that there was a reduction in the error of the models as the iterations of the genetic algorithm progressed with varying degrees of success across our different set-ups. Convergence was seen in both ramp and unloading/lower hysteresis error analysis with 20 and 50 iterations, respectively; however, there was still a significant reduction in the error of the model up to 50 iterations in the loading/upper hysteresis error calculations.

Focusing on the ramp section of the error analysis, it was seen to be the easiest for the algorithm to converge doing so in just over five iterations across all simulations. However, the hysteresis error was more evident across both loading and unloading and demonstrated the benefits of progressing the algorithm to 50 iterations. An *n* = 1 Prony series performs the best for the upper portion and the worst for the lower whereas *n* = 5 is the opposite. Both series report ~10% difference from best to worst performing iterations within the series, whereas the *n* = 3 Prony series may be the better overall solution resulting in ~5% cumulative error, supported by the standard deviation for these simulations (figure 6*e,f*). The different number of iterations investigation has enabled the evaluation of how many generations are needed to establish an accurate approximation of the optimization problem. Moreover, the error difference between models employing a different order, *N*, of the Prony series evidences how this can be exploited to improve predictions. Final mean errors were in the range of 15–30% for the best-performing outputs, across all types of tests: ramp and loading–unloading cycles. Although these errors may appear large, they incorporate the uncertainty as a consequence of variability in material properties in human AC. Furthermore, validation does not aim to directly match one dataset to one model or one test method, which can artificially reduce errors and can ignore natural variability. Instead, a training set is used in this study, as per practices in machine learning. It was also important that the optimization component had a low coding knowledge requirement to provide a framework more easily accessible to those interested in numerical modelling. This would allow it to be transferred for use in other applicable simulations with only minor adjustments to the code base; freely available [32].

The technique for model error calculation is seen to effectively provide a basis for the genetic algorithm to rank all the previous solutions as all simulations were able to progress and reduce the error by a meaningful amount after the initial optimization process. Part of what could have been hindering the algorithm in some instances is the variation that was seen in the initial dataset that was used to create the testing set. This resulted from the variation seen in the human AC tissue obtained and its varying level of damage and its donor’s age, which could have resulted in a less clear view of what the ‘ideal’ model should be. Narrowing this down in the future should reduce the errors measured for model results for a similar set of simulations; however, it does have its limitations owing to the difficulty in obtaining human tissue of the same standard. Further, filtering the dataset employed to constrain variability, by definition, limits the range of human AC which the model is trained to represent, and can induce artefacts associated with overfitting. Building a validation set of healthy/unhealthy tissue in the future would be beneficial to the development of models representing healthy/damaged cartilage tissue. In addition to this, building a much larger dataset would provide more points for the machine learning algorithm to use in its prediction of the parameters increasing the likelihood of a reduced error comparison to the tissue experimental data.

Prony series have been shown to be useful for characterizing biological tissues in the time domain, so as to predict frequency-domain mechanics [25,46]. However, a challenge with Prony series is that there is not one single set of parameters which may represent a dataset. Hence, a genetic algorithm has been employed in this study to identify the optimal parameters which best characterize a hyper-viscoelastic material and focus on error analysis. The rationale for presenting data for series with *n* = 1, 3 and 5 is also to aid in evidencing how a Prony series may vary with the series order, including how error may vary. Furthermore, the model error can be reduced by increasing the experimental dataset, particularly for training. In terms of numerical modelling, a separate approach could be to evaluate a wider range of material properties including some which are more specific as to the constitutive composition of the material. Separately, additional mutation algorithms that occur during the parameter creation portion of the genetic algorithm could be evaluated. However, it should noted that biological tissues can experience fracture strain [47]. In our current study, we have avoided tissue fracture by loading to an induced stress below 2 MPa; typically >2.8 MPa would be required to induce failure during cyclic loading of AC [48]. If fracture were to occur during testing, the characterization would include an inherent bias within the parameters optimized. The authors have targeted the widest possible application for frequency-domain measures to time-domain modelling; however, there is scope to tailor the code used to a given application further.

A limitation of the use of a Prony series to characterize the viscoelastic behaviour of AC is that it does not directly prescribe properties to the composition of AC. However, Cederlund & Aspden [49] have recently questioned some of the assumptions in the literature around the current understanding of the role of water in AC mechanics. Moreover, material properties of soft connective tissues can vary by orders of magnitude across studies [20]; for cartilage this may primarily be owing to rate [50] and magnitude of loading [23]. The model which is presented in this current study makes no assumptions as to the role of constituents in the physical behaviour of the material. In terms of clinical applications, the data can be seen as providing a standard against which replacement materials for AC can be evaluated; particularly if these are designed to mimic the dynamic behaviour of AC. Furthermore, this study presents a framework by which to ‘translate’ frequency-domain to time-domain data for hyper-viscoelastic materials, independent of the case study of AC. Hence, the framework and code provided have applications to synthetic materials which are not solely biomaterials.

The framework presented enables the use of frequency-domain test results ready for use within time-domain models, such as for FEA. Although Abaqus has been applied for the current study, the framework could be implemented with other FEA software that has scripted access to input files. Examples include FEBio and LS-Dyna which have previously been used for automatic spine [16] and heart valve [51] modelling, respectively. However, the interfacing scripts would require amending. The system is transferable in its use owing to the modular design of its components, though the intricacies of the parameter alteration during the optimization procedures require a problem-specific definition. Thus, it requires some simulation-specific knowledge for implementation to a different material. This would mainly involve changing the fitness characterization and optimization equations within the optimization control node as displayed in the system structure (figure 2).

## 5. Conclusion

This article presents a framework that allows the automation of simultaneously characterizing and validating hyper-viscoelastic material properties for a given material. The system used here can be useful in furthering the characterization of soft materials, not least biomaterials. Indeed, frequency-domain test data for AC have been used to produce the Prony series alongside a hyper-elastic material model which can be implemented in a time domain, including through FEA.

## Data Availability

Data and relevant code for this research work are stored in GitHub [52] and have been archived within the Zenodo repository [32]. Supplementary material is available online [53].
